# Comparative risk of incidence and clinical outcomes of COVID-19 among proton pump inhibitor and histamine-2 receptor antagonist short-term users: a nationwide retrospective cohort study

**DOI:** 10.1186/s40360-022-00549-7

**Published:** 2022-01-17

**Authors:** Jimyung Park, Seng Chan You, Jaehyeong Cho, Chan Hyuk Park, Woon Geon Shin, Rae Woong Park, Seung In Seo

**Affiliations:** 1 Department of Biomedical Sciences, Ajou University Graduate School of Medicine, 164, World cup-ro, Yeongtong-gu, Suwon-si, Gyeonggi-do 16499 Republic of Korea; 2 Department of Preventive Medicine, Yonsei University College of Medicine, Seoul, Republic of Korea; 3 Department of Internal Medicine, Hanyang University Guri Hospital, Hanyang University College of Medicine, Guri, Republic of Korea; 4 Division of Gastroenterology, Department of Internal Medicine, Kangdong Sacred Heart Hospital, Hallym University College of Medicine, 150, Seongan-ro, Seoul, Gangdong-gu 05355 South Korea; 5 Department of Biomedical Informatics, Ajou University School of Medicine, Suwon, Gyeonggi-do Republic of Korea

**Keywords:** Retrospective studies, Pharmacovigilance, Epidemiology, Acid suppressants

## Abstract

**Background:**

This study aimed to evaluate incidence risk and adverse clinical outcomes in COVID-19 disease among short-term users of acid-suppressants in South Korea.

**Methods:**

This retrospective cohort study, conducted using a nationwide claims database for South Korea, used data from patients with COVID-19 tested between January 1 and May 15, 2020. Patients aged over 18 years and prescribed proton pump inhibitors (PPI) or histamine-2 receptor antagonist (H_2_RA) for more than 7 days were identified. Primary outcome was COVID-19 while secondary outcomes were all-cause mortality, hospitalization with respiratory disease, or intensive respiratory intervention. Large-scale propensity scores were used to match patients, while the Cox proportional hazard model was utilized to evaluate any association between exposure and outcome(s). The risk estimates were calibrated by using 123 negative control outcomes.

**Results:**

We identified 26,166 PPI users and 62,117 H_2_RA users. After propensity score matching, compared to H_2_RA use, PPI use was not significantly associated with lower risk of COVID-19 (calibrated hazard ratio [HR], 0.81 [95% confidence interval (CI), 0.30–2.19]); moreover, PPI use was not associated with adverse clinical outcomes in COVID-19, namely, hospitalization with respiratory disease (calibrated HR, 0.88 [95% CI, 0.72–1.08]), intensive respiratory interventions (calibrated HR, 0.92 [95% CI, 0.46–1.82]), except for all-cause mortality (calibrated HR, 0.54 [95% CI, 0.31–0.95]).

**Conclusions:**

In this study, we found that the PPI user was not associated with risk of COVID-19 compared to H_2_RA users. There was no significant relationship between severe clinical outcomes of COVID-19 and exposure to PPI compared with H_2_RA, except for all-cause mortality.

**Supplementary Information:**

The online version contains supplementary material available at 10.1186/s40360-022-00549-7.

## Introduction

Proton pump inhibitors (PPI) are the mainstay in the management of acid-related gastrointestinal disease, including gastroesophageal reflux disease and peptic ulcer disease, and for the prevention of GI bleeding and stress ulcers [1]. While their widespread use has resulted in the improvement of acid-related disorders, concerns about potential complications due to PPI use, such as osteoporosis, dementia, malabsorption, gastrointestinal neoplasia, and increased susceptibility to bacterial infection, have also been rising [2, 3]. Further, it is possible that acid-suppressant drugs could increase susceptibility to respiratory infections because they counter the acidic environment of stomach, thereby allowing bacterial colonization [4, 5]. Even though several studies have evaluated the association between pneumonia and acid-suppressive drugs, the results remain inconclusive [4, 6–10].

Recently, several studies have described the effects of acid-suppressive agent use on the clinical course of and susceptibility to SARS-CoV-2 infection (COVID-19) [11–15]; however, few studies have directly compared incidence and risk of complications in COVID-19 between PPI and histamine-2 receptor antagonist (H_2_RA) therapy. In 2011, a systematic review and meta-analysis by Eom et al. investigated the association between use of acid-suppressive drugs and risk of pneumonia and found that the overall risk was higher among people using PPIs than H_2_RAs (adjusted odds ratio [OR] 1.27, 95% confidence interval [CI] 1.11–1.46 vs. 1.22, 95% CI 1.09–1.36) [7]. Nonetheless, the influence of acid-suppressive drugs on the viral pneumonia remains controversial.

To date, only limited data is available on the relationship between acid suppression therapy and clinical course of COVID-19 infection; therefore, we conducted a population-based retrospective cohort study to compare the risk of complications in COVID-19 among Korean patients prescribed PPI and H_2_RA therapy.

## Methods

### Data sources

A national claims database in South Korea that included COVID-19 testing data was used in this study [16]. The database was obtained from the Health Insurance Review and Assessment service (HIRA) which is the South Korean national institution for reviewing and assessing national health insurance claims. In response to the COVID-19 pandemic, HIRA collected data on COVID-19 testing by the reverse transcriptase polymerase chain reaction method from 1 January to 15 May, 2020. Notably, the collected data were converted into the Observational Medical Outcomes Partnership (OMOP) common data model (CDM), version 5, and released to the public. Hospitalization records were extracted for all patients involved in the study. This study was approved informed consent waiver by the institutional review board of the Kangdong Sacred Heart Hospital (no. 2020–04-001). All methods were carried out in accordance with relevant guidelines and regulations.

### Study population and exposure

We identified patients aged over 18 years and diagnosed with COVID-19 disease. The cohort comprised patients prescribed acid-suppressants for 7 days or more. The index date was defined as the first day of drug treatment. The PPIs prescribed were defined as rabeprazole, pantoprazole, omeprazole, lansoprazole, ilaprazole, esomeprazole, and dexlansoprazole, while the H_2_RAs were defined as ranitidine, nizatidine, lafutidine, famotidine, and cimetidine. We excluded all patients prescribed any other primary or secondary medication(s) (i.e., PPIs and H_2_RAs) within 180 days before the index date. We defined the drug exposures as continuous exposure if the date gap between drug prescriptions was less than 30 days. Acid-suppressant non-users were defined as the patients who were not prescribed acid-suppressants and were not diagnosed with COVID-19 within 180 days before the index date.

### Outcomes

The primary outcome was defined as diagnosis of COVID-19 and the secondary outcomes were defined as the complications of COVID-19, namely, (1) all-cause mortality, (2) hospitalization with at least one of the following diagnoses, i.e., pneumonia, acute respiratory disease syndrome (ARDS), sepsis, or acute kidney injury (AKI), and (3) requirement of intensive respiratory interventions such as mechanical ventilation, extracorporeal membrane oxygenation procedure (ECMO), or tracheostomy.

### Statistical analyses

We used large-scale propensity score matching (PSM) with regularized logistic regression models to balance baseline characteristics of the study cohorts [17, 18]. Three different methods were used in the analysis, (1) propensity score unadjusted analysis, (2) one-to-four exact PSM with greedy nearest method, and (3) propensity score stratification with five strata [19]. The covariates included age, sex, all medication(s), medical procedure(s), disease history, and comorbidity index in the database. Cox proportional hazards regression models were used to estimate the association between exposures and outcomes. Patients were censored if they were no longer observable in the database. Data analyses were performed for three different cohort comparisons— (1) PPI users versus H_2_RA users, (2) PPI users versus non-users, and (3) H_2_RA users versus non-users.

During secondary analysis, only patients with a definite diagnosis of COVID-19 were included to measure complications due to COVID-19 disease among infected patients who had been prescribed acid-suppressive agents (i.e., PPIs and H_2_RAs). Moreover, we added the hospitalization criteria to investigate the clinical outcomes among COVID-19 patients with severe symptoms. Other analysis settings were identical to that used in primary analysis, i.e., PPI users, H_2_RA users, and non-users. Overall, six different cohort settings were applied in the secondary analysis (3 analyses among COVID-19 groups + 3 analyses among hospitalized COVID-19 groups).

Even though we utilized large-scale propensity score matching to balance between study groups and to minimize the unmeasured confounders, there still can be the residual bias in the observational studies [18]. To estimate the systematic error in the models, we employed 123 negative control outcomes to estimate systematic error in the models (Supplementary Table 1) [20, 21]. The negative control outcomes were found not to be affected by acid-suppressant use, hence, the negative control outcomes can show whether the model is influenced by unmeasured confounders or not. In this study, the final hazard ratio (HR) and 95% CIs were reported through empirical calibrations to adjust measured systematic errors from the analysis of 123 negative control outcomes [22].

## Results

### PPI use and risk of COVID-19

#### Baseline characteristics of the study population

**Fig. 1:**
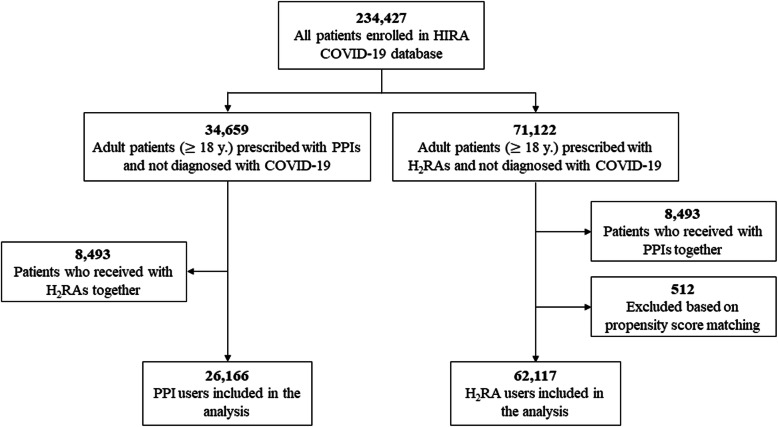
Flowchart in the primary analysis of PPI use and risk of COVID 19

Of the 234,427 patients in the HIRA COVID-19 database, we finally included 26,166 patients prescribed PPI and 62,117 patients prescribed H_2_RA (Fig. 1) with a person-years follow-up duration of 2361 days for PPI users and 3674 days for H_2_RA users. Median follow-up days for PPI users was 14 (interquartile range [IQR], 7–75) while it was 7 for H_2_RA users (IQR, 7–34). Baseline characteristics of the primary analysis are listed in Table 1**,** which also provides standardized mean differences before and after PSM for the study population. Overall, 20,202 covariates were used for matching (Supplementary Fig. 1), and among overall matched covariates, absolute standardized differences after PSM were less than 0.1 for 96.04% of the covariates, implying that the cohorts were adequately matched and therefore, comparable. Covariates with standardized differences greater than 0.1 after PSM were predominantly medications associated with acid-related disorders and anti-inflammatory products (e.g., bismuth oxide and sucralfate).

**Table 1:** Baseline characteristics of patients with PPI and H_2_RA in the primary analysis

Characteristics, %	Before propensity score matching			After propensity score matching		
	PPI users (*n* = 26,166)	H_2_RA users (*n* = 62,629)	Absolute standardized difference	PPI users (*n* = 26,166)	H_2_RA users (*n* = 62,117)	Absolute standardized difference
Age group, %						
18–19	0.9	1.8	0.08	1.3	1.6	0.02
20–24	4.7	8.3	0.15	6.5	7.4	0.03
25–29	6.7	9.3	0.10	8.3	8.6	0.01
30–34	6.3	8.0	0.07	7.7	7.4	0.01
35–39	8.0	8.5	0.02	8.6	8.3	0.01
40–44	7.9	6.7	0.05	7.3	7.0	0.01
45–49	8.9	7.6	0.05	8.2	8.0	0.01
50–54	8.8	7.2	0.06	7.9	7.6	0.01
55–59	8.9	7.9	0.04	8.3	8.3	< 0.01
60–64	8.3	7.1	0.04	7.5	7.5	< 0.01
65–69	7.0	5.8	0.05	6.2	6.1	< 0.01
70–74	6.5	5.4	0.05	5.7	5.7	< 0.01
75–79	6.9	6.3	0.02	6.5	6.4	< 0.01
80–84	5.8	5.7	< 0.01	5.6	5.7	< 0.01
85–89	3.2	3.1	0.01	3.1	3.1	< 0.01
Gender, female, %	49.6	53.8	0.08	52.0	52.7	0.02
Medical history: general, %						
Acute respiratory disease	49.2	56.9	0.16	49.5	56.7	0.15
Chronic liver disease	5.6	3.9	0.08	5.0	4.0	0.05
Chronic obstructive lung disease	4.6	3.7	0.05	4.1	3.9	0.01
Depressive disorder	12.3	11.9	0.01	11.5	12.1	0.02
Diabetes mellitus	17.4	14.6	0.08	15.8	15.1	0.02
Gastrointestinal hemorrhage	3.5	1.9	0.10	3.3	1.9	0.09
Hyperlipidemia	38.6	30.9	0.16	35.7	31.7	0.08
Hypertensive disorder	35.4	29.7	0.12	35.7	31.7	0.08
Lesion of liver	4.7	3.0	0.09	4.2	3.2	0.06
Osteoarthritis	11.5	15.9	0.13	10.9	16.1	0.15
Pneumonia	7.4	6.3	0.04	6.6	6.6	< 0.00
Renal impairment	7.2	3.7	0.16	6.2	3.8	0.11
Rheumatoid arthritis	2.2	2.2	0.00	2.0	2.2	0.02
Ulcerative colitis	0.2	0.2	< 0.01	0.2	0.2	< 0.01
Atrial fibrillation	2.6	1.5	0.07	2.3	1.6	0.05
Cerebrovascular disease	5.2	4.8	0.02	4.7	4.9	0.01
Heart disease	18.6	12.7	0.16	16.8	13.1	0.10
Heart failure	7.9	4.8	0.12	7.0	5.1	0.08
Ischemic heart disease	10.1	6.5	0.13	9.0	6.7	0.08
Peripheral vascular disease	9.9	9.6	0.01	9.0	9.8	0.03
Malignant neoplastic disease	9.0	7.6	0.05	8.1	8.0	< 0.01
Medication use, %						
Antibacterials for systemic use	58.2	69.5	0.24	57.4	69.4	0.25
Antidepressants	12.9	12.5	0.01	12.0	12.8	0.02
Antiepileptics	11.1	12.2	0.04	9.9	12.6	0.09
Anti-inflammatory and antirheumatic products	49.2	68.4	0.40	49.2	68.2	0.39
Antineoplastic agents	3.2	2.6	0.40	2.8	2.8	< 0.00
Antithrombotic agents	38.4	46.1	0.16	36.9	46.3	0.19
Diuretics	16.0	13.0	0.08	14.4	13.5	0.03
Drugs for obstructive airway disease	17.8	21.1	0.08	17.2	21.4	0.11
Drugs used in diabetes	16.2	12.9	0.09	14.6	13.4	0.03
Immunosuppressants	3.5	2.6	0.05	3.1	2.6	0.03
Lipid modifying agents	27.9	21.3	0.16	25.3	22.0	0.08
Opioids	50.4	61.3	0.22	29.2	61.6	0.25
Charlson comorbidity index	2.79	2.79	0.17	2.32	2.61	0.06

Values are presented as proportion of the patients (%)

*Abbreviation*: *PPI* proton pump inhibitor, *H*_*2*_*RA* histamione-2 receptor antagonist

#### Association of PPI use and risk of COVID-19

Table 2 shows the results of the primary analysis that estimated the association between PPI or H_2_RA usage and risk of COVID-19. PSM-unadjusted analysis showed that PPI use was not significantly associated with risk of COVID-19 infection compared to H_2_RA use (calibrated HR, 1.49 [95% CI, 0.66–3.36]), moreover, PSM adjusted analyses revealed that PPI use was not significantly associated with lower risk of COVID-19 infection in one-to-four PSM analysis (calibrated HR, 0.81 [95% CI, 0.30–2.19]) and in stratification of propensity scores analysis (calibrated HR, 1.03 [95% CI, 0.51–2.08]).

**Table 2:** The associations of the risk of COVID-19 infection between PPI and H_2_RA users

Analysis settings	No. of subjects		No. of outcome occurrence		calibrated HR [95% CI]
	PPI users	H_2_RA users	PPI users	H_2_RA users	
Unadjusted	26,166	62,629	96	104	1.49 (0.66–3.36)
Stratification	26,166	62,629	96	104	1.03 (0.51–2.08)
1:4 matching	26,166	62,117	96	104	0.81 (0.30–2.19)
Analysis settings	No. of subjects		No. of outcome occurrence		calibrated HR [95% CI]
	PPI users	Non-users	PPI users	Non-users	
Unadjusted	26,044	74,975	113	3012	0.43 (0.11–1.61)
Stratification	26,044	74,975	113	3012	0.50 (0.17–1.52)
1:4 matching	22,765	71,408	111	2848	0.47 (0.17–1.29)
Analysis settings	No. of subjects		No. of outcome occurrences		calibrated HR [95% CI]
	H_2_RA users	Non-users	H_2_RA users	Non-users	
Unadjusted	51,545	64,013	112	2616	0.30 (0.09–0.96)
Stratification	51,545	64,013	112	2616	0.46 (0.15–1.43)
1:4 matching	29,845	64,013	107	2616	0.48 (0.17–1.37)

*Abbreviation*: *PPI* proton pump inhibitor, *H*_*2*_*RA* histamione-2 receptor antagonist, *HR* hazard ratio, *CI* confidence interval

A comparison of COVID-19 between acid-suppressant users and non-users revealed that PPI use was not significantly associated with lower risk of COVID-19 compared to non-users before adjusted analysis (calibrated HR, 0.43 [95% CI, 0.11–1.61]), after one-to-four matched analysis (calibrated HR, 0.47 [95% CI, 0.17–1.29]), and stratification analysis (calibrated HR, 0.50 [95% CI, 0.17–1.52]). Among H_2_RA users, medication was associated with the lower risk of COVID-19 compared to non-users during unadjusted analysis (calibrated HR, 0.30 [95% CI, 0.09–0.96]). In other analyses, H_2_RA use was not significantly associated with infection despite one-to-four PSM (calibrated HR, 0.48 [95% CI, 0.17–1.37]) or propensity score stratification (calibrated HR, 0.46 [95% CI, 0.15–1.43]).

### PPI use and complications of COVID-19 disease

#### Baseline characteristics of the study population

**Fig. 2:**
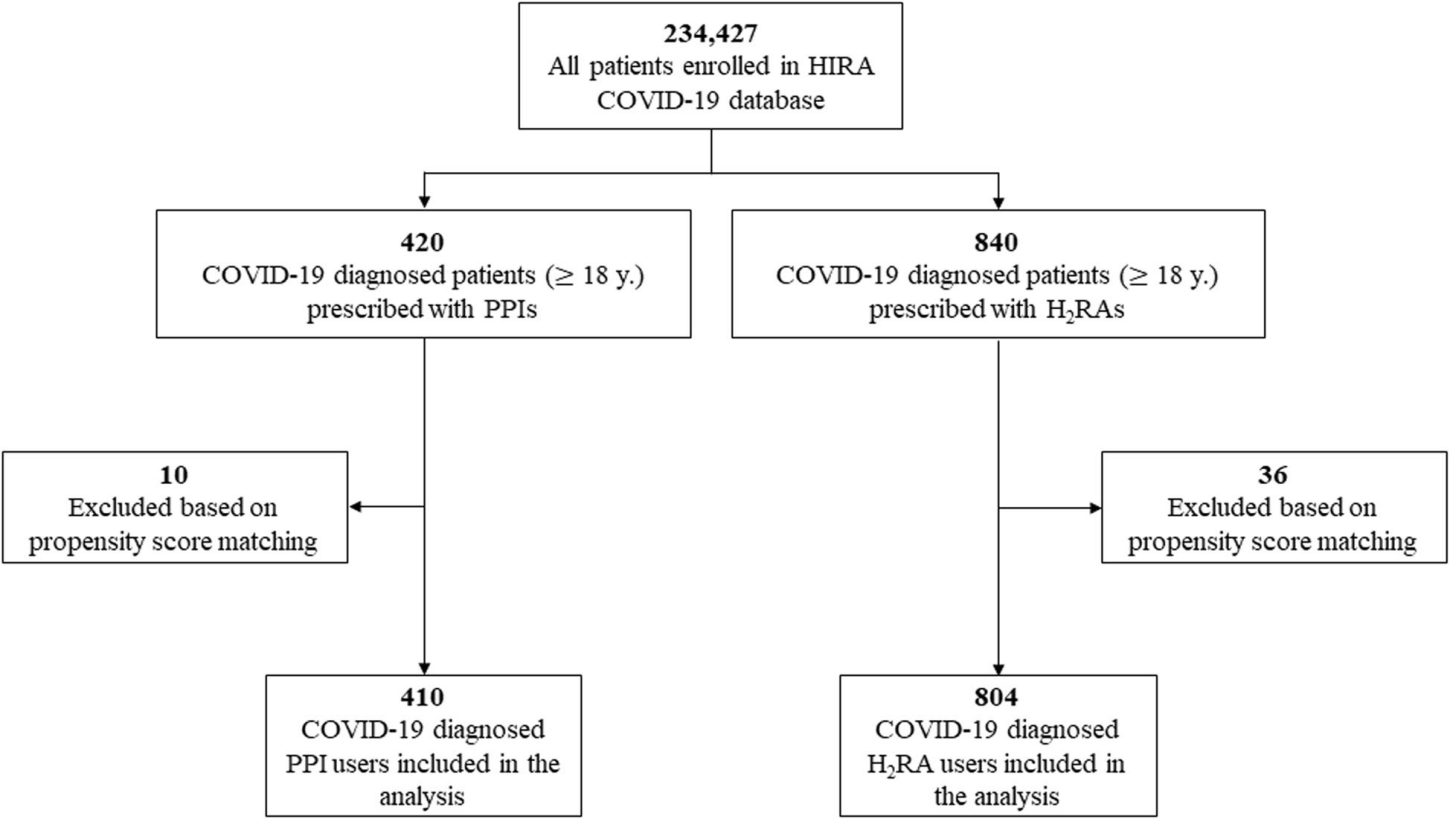
Flowchart in the secondary analysis of PPI use and complications of COVID-19 disease

Secondary analysis was performed with data from 1260 patients diagnosed with COVID-19; of these, 410 patients were prescribed PPI and 804 were given H_2_RA medication (Fig. 2). Subjects were matched based on sex, age groups, medical history (chronic obstructive lung disease and chronic kidney disease), and the Charlson comorbidity index, and the absolute standardized mean difference for all covariates after PSM was less than 0.1. Baseline characteristics are presented in Table 3.

**Table 3:** Baseline characteristics of COVID-19 patients with PPI and H_2_RA in the secondary analysis

Characteristics, %	Before propensity score matching			After propensity score matching		
	PPI users (*n* = 420)	H_2_RA users (*n* = 840)	Absolute standardized difference	PPI users (*n* = 410)	H_2_RA users (*n* = 804)	Absolute standardized difference
Age group, %						
18–19	< 1.2	1.7	0.06	< 1.2	1.7	0.06
20–24	4.8	10.4	0.21	4.9	4.8	0.01
25–29	4.5	8.1	0.15	4.6	4.3	0.02
30–34	4.0	2.9	0.06	4.1	4.0	0.01
35–39	2.6	7.0	0.21	2.7	2.8	0.01
40–44	3.1	5.2	0.11	3.2	3.7	0.03
45–49	5.7	7.5	0.07	5.9	4.7	0.05
50–54	12.1	8.3	0.13	12.2	11.2	0.03
55–59	12.1	10.7	0.04	12.4	12.2	0.01
60–64	16.7	10.1	0.19	16.3	13.1	0.09
65–69	10.2	7.3	0.10	10.0	9.6	0.01
70–74	6.9	6.4	0.02	6.8	7.8	0.04
75–79	7.1	6.5	0.02	7.3	8.9	0.06
80–84	4.5	3.8	0.04	4.4	5.2	0.04
85–89	3.3	3.0	0.02	2.9	4.4	0.08
Gender: female, %	60.7	66.8	0.08	61.0	65.9	0.02
Medical history: general, %						
Chronic obstructive lung disease	< 0.1	< 0.1	< 0.1	< 0.1	0.12	0.09
Chronic kidney disease	< 0.1	< 0.1	0.11	< 0.1	< 0.1	0.09
Charlson comorbidity index	3.0	2.3	0.27	3.0	2.7	0.1

Values are presented as proportion of the patients (%)

*Abbreviation*: *COVID-19* SARS-CoV-2 infection, *PPI* proton pump inhibitor, *H*_*2*_*RA* histamione-2 receptor antagonist

#### Association between PPI use and complications of COVID-19 disease

Table 4 provides the results of the secondary analysis, which showed no significant association between PPI or H_2_RA use and all-cause mortality among COVID-19 patients, i.e., (1) unadjusted analysis (calibrated HR, 0.82 [95% CI, 0.47–1.41]), or (2) propensity score stratification (calibrated HR, 0.63 [95% CI, 0.37–1.07]), however, (3) one-to-four PSM analysis showed significant associated between PPI use and all-cause mortality (calibrated HR, 0.54 [95% CI, 0.31–0.95]). Further, hospitalization with pneumonia, ARDS, sepsis, or AKI were not associated with PPI or H_2_RA use, irrespective of the type of analysis, i.e., (1) unadjusted analysis (calibrated HR, 1.12 [95% CI, 0.87–1.43]), (2) propensity score stratification (calibrated HR, 0.96 [95% CI, 0.79–1.17]), or (3) one-to-four PSM (calibrated HR, 0.88 [95% CI, 0.72–1.08]). Similarly, there was no association between PPI and requirement for intensive respiratory interventions, (1) unadjusted analysis (calibrated HR, 1.28 [95% CI, 0.65–2.50]), (2) propensity score stratification (calibrated HR, 1.01 [95% CI, 0.52–1.97]), and (3) one-to-four PSM (calibrated HR, 0.92 [95% CI, 0.46–1.82]). The results for hospitalized COVID-19 patients are provided in Table 5.

**Table 4:** The associations of the clinical outcomes between PPI and H_2_RA users among COVID-19 diagnosed patients

Outcome	Analysis settings	No. of subjects		No. of outcome occurrence		calibrated HR [95% CI]
		PPI users	H_2_RA users	PPI users	H_2_RA users	
All-cause mortality	Unadjusted	420	840	21	40	0.82 (0.47–1.41)
	Stratification	420	840	21	40	0.63 (0.37–1.07)
	1:4 matching	410	804	19	40	0.54 (0.31–0.95)
Outcome	Analysis settings	No. of subjects		No. of outcome occurrence		calibrated HR [95% CI]
		PPI users	H_2_RA users	PPI users	H_2_RA users	
Hospitalization with pneumonia, ARDS, sepsis, and AKI	Unadjusted	393	789	164	262	1.12 (0.87–1.43)
	Stratification	393	789	164	262	0.96 (0.79–1.17)
	1:4 matching	382	754	160	259	0.88 (0.72–1.08)
Outcome	Analysis settings	No. of subjects		No. of outcome occurrence		calibrated HR [95% CI]
		PPI users	H_2_RA users	PPI users	H_2_RA users	
Occurrence of a composite intensive respiratory intervention	Unadjusted	411	831	16	20	1.28 (0.65–2.50)
	Stratification	411	831	16	20	1.01 (0.52–1.97)
	1:4 matching	402	795	15	20	0.92 (0.46–1.82)

*Abbreviation*: *COVID-19* SARS-Cov-2, *PPI* proton pump inhibitor, *H*_*2*_*RA* histamione-2 receptor antagonist, *HR* hazard ratio, *CI* confidence interval, *ARDS* acute respiratory distress syndrome, *AKI* acute kidney injury

**Table 5:** The associations of the clinical outcomes between PPI and H_2_RA users among hospitalized patients due to COVID-19

Outcome	Analysis settings	No. of subjects		No. of outcome occurrence		calibrated HR [95% CI]
		PPI users	H_2_RA users	PPI users	H_2_RA users	
All-cause mortality	Unadjusted	371	747	12	30	0.60 (0.30–1.20)
	Stratification	371	747	12	30	0.50 (0.26–0.99)
	1:4 matching^a^	366	719	11	30	0.52 (0.26–1.06)
Outcome	Analysis settings	No. of subjects		No. of outcome occurrence		calibrated HR [95% CI]
		PPI users	H_2_RA users	PPI users	H_2_RA users	
Occurrence of a composite intensive respiratory intervention	Unadjusted	364	737	10	12	1.28 (0.54–3.04)
	Stratification	364	737	10	12	1.10 (0.47–2.58)
	1:4 matching^a^	358	709	9	12	0.98 (0.40–2.39)

*Abbreviation*: *PPI* proton pump inhibitor, *H*_*2*_*RA* histamione-2 receptor antagonist, *HR* hazard ratio, *CI* confidence interval

^a^The propensity matching in secondary analysis included only age, sex, and comorbidity indices

## Discussion

This study aimed to estimate and compare risk of incidence and assess outcomes after COVID-19 in Korean patients prescribed PPI or H_2_RA. Specifically, we evaluated the incidence of COVID 19 in subjects prescribed PPI or H_2_RA for more than 7 days and show that short-term use of PPI was not associated with incidence of COVID-19 compared to short-term H_2_RA users or non-users. Moreover, among COVID-19 patients, PPI use for ≥ 7 days was not significantly associated with risk of complications compared to H_2_RA use except for all-cause mortality in PSM analysis, and it was not associated with complications in hospitalized patients.

Two recent studies have addressed the association between PPI use and incidence of COVID-19 infection [11, 12]. Lee et al. have reported that patients taking PPIs are at increased risk for severe clinical outcomes with COVID-19 but that they are not more susceptible to SARS- CoV-2 infection [12]. They defined current PPI users as patients who took PPIs 1–30 days before the first SARS-CoV-2 test date [12]. Further, they also showed that there was no significant difference in SARS-CoV-2 positivity rates between PPI users and non-users, irrespective of short-term (< 30 days) or long-term (> 30 days) use [12]. Another study, an online survey by Almario et al., reported that individuals using PPIs up to once daily (aOR 2.15; 95%CI, 1.90–2.44) or twice daily (aOR 3.67; 95% CI, 2.93–4.60) had significantly higher odds for testing COVID-19 positive compared to those not taking PPIs [11]. In contrast, we show that PPI use was not associated with the higher risk of COVID-19 infection compared to H_2_RA use or no acid-suppressant use. This could be due to our use of the Korean national claims database wherein data was converted to the OMOP-CDM format, and this permitted adjustment for many more covariates than previous studies. Additionally, large-scale propensity matching was used to overcome potentially unmeasured confounding factors and we also performed multiple sensitivity analyses. We also calibrated our analysis using 123 negative control outcomes to detect and reduce confounding factors, selection bias, and systematic errors. Thus, of the 48 analyses performed (9 primary and 39 secondary), most results were consistent with the calibrations.

In the secondary analysis, we compared the complication of COVID-19 between PPI and H_2_RA using multiple sensitivity analyses. The result showed no significant association between PPI and H_2_RA. Only PSM analysis measuring the association between PPI and all-cause mortality, compared to H_2_RA, showed significant results, however, the other analyses (i.e., unadjusted and stratification) showed opposite results. We could not perform large-scale PSM in the secondary analysis due to small number of included COVID-19 patients, therefore, there might be biases in the result.

To date, several studies have addressed clinical outcomes in COVID-19; however, most studies only included a small number of patients and were limited by the presence of confounding factors [12–14, 23]. Lee et al. found that PPI use led to greater risk of severe clinical outcomes in COVID-19, including intensive care unit admission, requirement of invasive ventilation, or death [12]. However, that study did not consider PPI use after COVID-19 diagnosis, and the comparator group comprised non-PPI users, which could have led to indication bias, i.e., patients in the PPI group could have experienced a more severe course of COVID-19 compared to non-users and the difference might have led to more severe outcomes. Therefore, to avoid indication bias, we compared clinical outcomes between PPI and H_2_RA users, and consistent with our results, Zhang et al. also reported that PPI use had no effect on the clinical course of COVID-19 [13]. Additionally, Taştemur et al. have suggested that PPIs may be used for both prophylaxis and treatment because hydroxychloroquine and azithromycin may prevent viral spread by accumulating in organelles with acidic content and raising their pH. Thus, given their effects on pH, they concluded that PPIs show similar effects on viral entry and intracellular distribution [15]. Such inconsistent results imply that the risk and benefits of PPI use in viral infection have remained controversial to date [23].

Our study has certain limitations. First, although we used large-scale PSM in the primary analysis, there were a few relatively unmatched covariates that may have affect the results, showing standardized mean difference greater than 0.1. Nonetheless, we measured and adjusted the systematic error in this study through empirical calibration by employing 123 negative control outcomes in the primary analysis. In the secondary analysis, we could not perform large-scale PSM, therefore, the results of clinical outcomes might have many biases. Second, we only included PPI use for 7 days, and therefore, we could not evaluate the effects of long-term PPI use, and as the HIRA database also had data only pertaining to a short period, we could not analyze the long-term effects of acid-suppressants. Third, this was an observational study; therefore, it is not possible to establish causality. Although we could not perform well-designed randomized controlled trial, we performed large-scale PSM and analyzed negative control outcomes to adjust systematic unmeasured confounding factors. Nonetheless, the effects of acid-suppressants on viral infection, especially COVID-19, require further clarification.

## Conclusions

In this study, using large-scale PSM and multiple sensitivity analyses, we show that, compared to H_2_RA use, short-term PPI use is not associated with incidence of COVID-19 infection and severe clinical outcomes. Nevertheless, the effects of long-term PPI use on the incidence and clinical outcomes in COVID-19 disease need to be clearly established.

## Supplementary Information

**Additional file 1.**

## Data Availability

The data is not publicly available. All data generated or analysed during this study are included in this published article and its supplementary information files.

## Abbreviations

PPI: Proton pump inhibitor
COVID-19: SARS-CoV-2 infection
H_2_RA: Histamine-2 receptor antagonist
OR: Odds ratio
CI: Confidence interval
HIRA: Health Insurance Review and Assessment
OMOP: Observation Medical Outcomes Partnership
CDM: Common Data Model
ARDS: Acute respiratory disease syndrome
AKI: Acute kidney injury
ECMO: Extracorporeal membrane oxygenation
PSM: Propensity score matching
HR: Hazard ratio
IQR: Interquartile range
