# Supplementary material for: Comparative risk of incidence and clinical outcomes of COVID-19 among proton pump inhibitor and histamine-2 receptor antagonist short-term users: a nationwide retrospective cohort study
Source: BMC Pharmacol Toxicol. 2022 Jan 17;23:9. doi: 10.1186/s40360-022-00549-7 (PMC8762632; doi:10.1186/s40360-022-00549-7)
Supplement: Supplementary file 1 — Additional file 1. [file 40360_2022_549_MOESM1_ESM.docx]

Supplementary Figure 1. Covariate balance plot showing the standardized mean differences of before and after propensity score matching in the primary analysis.


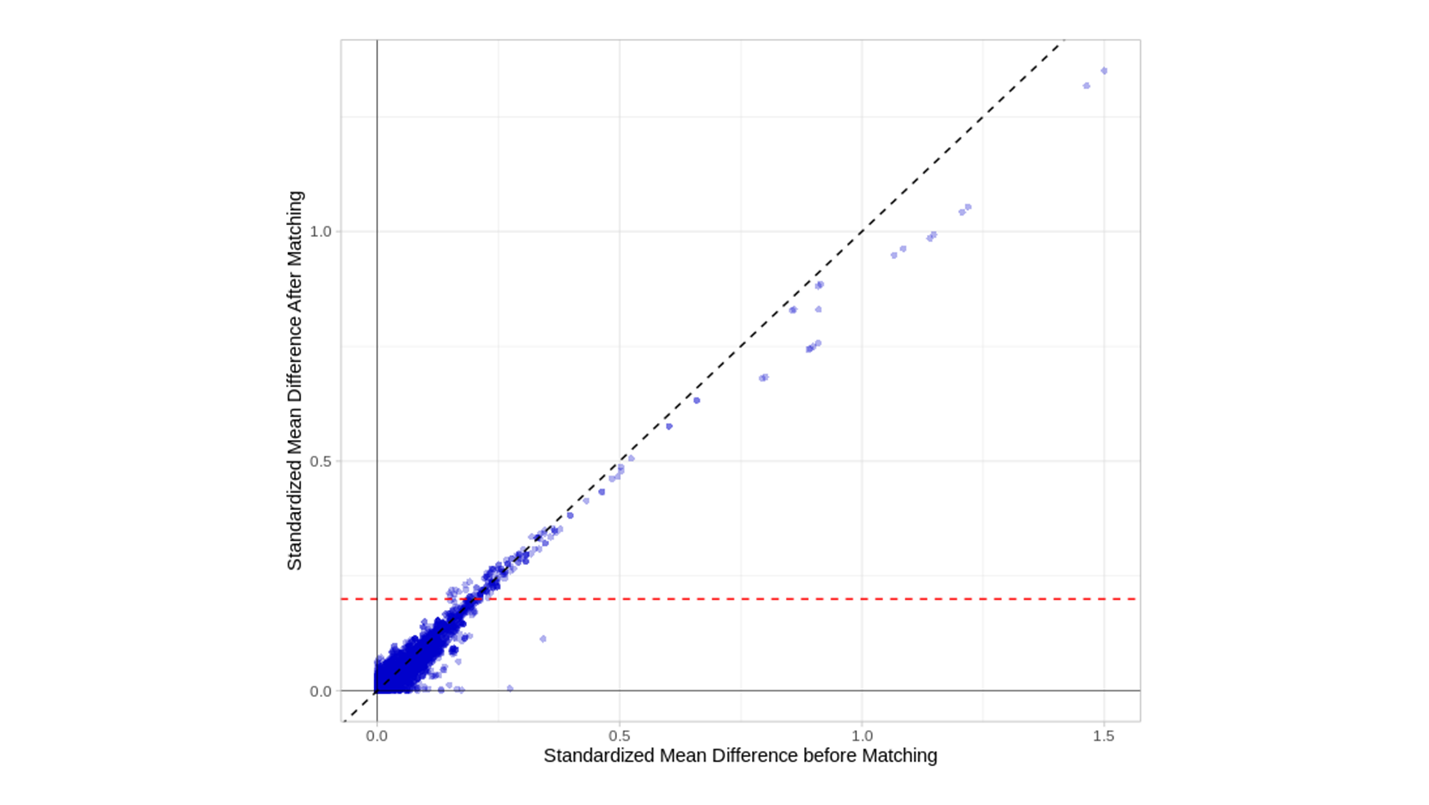


Supplementary Table 1. List of Negative Control Outcomes

| Abnormal cervical smear |
| --- |
| Abnormal pupil |
| Abrasion and/or friction burn of trunk without infection |
| Absence of breast |
| Absent kidney |
| Acariasis |
| Acid reflux |
| Acquired hallux valgus |
| Acquired keratoderma |
| Acquired trigger finger |
| Acute conjunctivitis |
| Amputated foot |
| Amyloidosis |
| Anal and rectal polyp |
| Ankylosing spondylitis |
| Aseptic necrosis of bone |
| Astigmatism |
| Bell's palsy |
| Benign epithelial neoplasm of skin |
| Burn of forearm |
| Calcaneal spur |
| Cannabis abuse |
| Cervical somatic dysfunction |
| Chalazion |
| Changes in skin texture |
| Chondromalacia |
| Chondromalacia of patella |
| Cocaine abuse |
| Colostomy present |
| Complication due to Crohn's disease |
| Contact dermatitis |
| Contusion of knee |
| Crohn's disease |
| Croup |
| Derangement of knee |
| Diabetic oculopathy |
| Difficulty sleeping |
| Disproportion of reconstructed breast |
| Effects of hunger |
| Endocarditis |
| Endometrial hyperplasia |
| Endometriosis |
| Enthesopathy |
| Epicondylitis |
| Epidermoid cyst |
| Epstein-Barr virus disease |
| Feces contents abnormal |
| Foreign body in orifice |
| Fracture of upper limb |
| Gallstone |
| Ganglion cyst |
| Genetic predisposition |
| Genital herpes simplex |
| Hammer toe |
| Hemangioma |
| Hereditary thrombophilia |
| Herpes zoster without complication |
| High risk sexual behavior |
| Hodgkin's disease |
| Homocystinuria |
| Human papilloma virus infection |
| Hypoglycemic coma |
| Hypopituitarism |
| Ileostomy present |
| Impacted cerumen |
| Impetigo |
| Impingement syndrome of shoulder region |
| Ingrowing nail |
| Injury of knee |
| Iridocyclitis |
| Irregular periods |
| Irritable bowel syndrome |
| Kwashiorkor |
| Late effect of contusion |
| Late effect of motor vehicle accident |
| Lesion of cervix |
| Leukorrhea |
| Lyme disease |
| Macular drusen |
| Malignant neoplasm of endocrine gland |
| Melena |
| Mononeuropathy |
| Nicotine dependence |
| Noise effects on inner ear |
| Nonspecific tuberculin test reaction |
| Non-toxic multinodular goiter |
| Onychomycosis |
| Onychomycosis due to dermatophyte |
| Opioid abuse |
| Osteochondropathy |
| Paraplegia |
| Passing flatus |
| Polyp of intestine |
| Postviral fatigue syndrome |
| Presbyopia |
| Problem related to lifestyle |
| Psychalgia |
| Ptotic breast |
| Pulmonary tuberculosis |
| Rectal mass |
| Regular astigmatism |
| Sarcoidosis |
| Scar |
| Senile hyperkeratosis |
| Septic shock |
| Sjögren's syndrome |
| Somatic dysfunction of lumbar region |
| Splinter of face, without major open wound |
| Sprain of ankle |
| Strain of rotator cuff capsule |
| Tear film insufficiency |
| Tietze's disease |
| Tobacco dependence syndrome |
| Tonsillitis |
| Toxic goiter |
| Ulcerative colitis |
| Vaginitis and vulvovaginitis |
| Verruca vulgaris |
| Viral conjunctivitis |
| Viral hepatitis |
| Visceroptosis |
| Wrist joint pain |
| Wristdrop |
